# Detection and Validation of Organic Metabolites in Urine for Clear Cell Renal Cell Carcinoma Diagnosis

**DOI:** 10.3390/metabo14100546

**Published:** 2024-10-13

**Authors:** Kiana L. Holbrook, George E. Quaye, Elizabeth Noriega Landa, Xiaogang Su, Qin Gao, Heinric Williams, Ryan Young, Sabur Badmos, Ahsan Habib, Angelica A. Chacon, Wen-Yee Lee

**Affiliations:** 1 Department of Chemistry and Biochemistry, University of Texas at El Paso, El Paso, TX 79968, USA; kholbrook@miners.utep.edu (K.L.H.); enoriegalanda2@utep.edu (E.N.L.); sobadmos@utep.edu (S.B.); ahabib@miners.utep.edu (A.H.); aachacon3@miners.utep.edu (A.A.C.); 2 Division of Health Services and Outcomes Research, Children’s Mercy Kansas City, Kansas City, MO 64108, USA; gequaye@cmh.edu; 3 Department of Mathematical Sciences, University of Texas at El Paso, El Paso, TX 79968, USA; xsu@utep.edu; 4 Biologics Analytical Operations, Gilead Sciences Incorporated, Oceanside, CA 94404, USA; qin.gao1@gilead.com; 5 Department Urology, Geisinger Clinic, Danville, PA 17822, USA; hwilliams1@geisinger.edu (H.W.); ryoung01@som.geisinger.edu (R.Y.)

**Keywords:** VOCs, ccRCC, urinary, metabolomics, diagnostic model, GC-MS, stir-bar sorptive extraction, renal cancer carcinoma

## Abstract

Background: Clear cell renal cell carcinoma (ccRCC) comprises the majority, approximately 70–80%, of renal cancer cases and often remains asymptomatic until incidentally detected during unrelated abdominal imaging or at advanced stages. Currently, standardized screening tests for renal cancer are lacking, which presents challenges in disease management and improving patient outcomes. This study aimed to identify ccRCC-specific volatile organic compounds (VOCs) in the urine of ccRCC-positive patients and develop a urinary VOC-based diagnostic model. Methods: This study involved 233 pretreatment ccRCC patients and 43 healthy individuals. VOC analysis utilized stir-bar sorptive extraction coupled with thermal desorption gas chromatography/mass spectrometry (SBSE-TD-GC/MS). A ccRCC diagnostic model was established via logistic regression, trained on 163 ccRCC cases versus 31 controls, and validated with 70 ccRCC cases versus 12 controls, resulting in a ccRCC diagnostic model involving 24 VOC markers. Results: The findings demonstrated promising diagnostic efficacy, with an Area Under the Curve (AUC) of 0.94, 86% sensitivity, and 92% specificity. Conclusions: This study highlights the feasibility of using urine as a reliable biospecimen for identifying VOC biomarkers in ccRCC. While further validation in larger cohorts is necessary, this study’s capability to differentiate between ccRCC and control groups, despite sample size limitations, holds significant promise.

## 1. Introduction

Renal (kidney) cancer is a heterogeneous disease that is comprised of several subtypes, each of which is associated with a unique natural histology and prognosis [1,2]. Approximately 81,610 new kidney cancer cases and 14,390 deaths are anticipated in the United States in 2024 [3]. It ranks as the sixth most common cancer in men (5%) and the ninth most common cancer in women (3%) in the US [3,4]. The rise in incidence is possibly due to the increased use of cross-sectional imaging studies in the evaluation of abdominal-related complaints [5,6]. Most kidney cancers are renal cell carcinomas (RCCs), where clear cell renal cell carcinoma (ccRCC) is the most common subtype, accounting for 70–80% of all kidney cancers. This histological description is due to the high lipid content in the cytoplasm dissolved during immunohistochemical preparation methods, leaving a clear cytoplasm appearance [7].

The biology of ccRCC is characterized by increased glucose uptake and glycolysis through the canonical Warburg effect [8]. In addition, reductive carboxylation is achieved through a glutamate-dependent pathway that involves the backward flow of the tricarboxylic acid cycle [9]. Fatty acid metabolism in ccRCC favors increased lipid synthesis and decreased β-oxidation [10]. This metabolic reprogramming of normal cellular pathways provides ccRCC tumor cells with the ability to survive in hypoxic and nutrient-deficient environments [11]. Understanding these metabolic abnormalities in ccRCC opens avenues for targeted discovery of diagnostic biomarkers [12,13,14,15].

Numerous ccRCC biomarkers have been developed, but to date, none has been approved for clinical use [16,17]. Therefore, there remains an unmet need to identify more ideal ccRCC biomarkers. Recent studies found that trained dogs could distinguish between individuals with and without cancers only by sniffing their urine with high sensitivity and specificity for patients with prostate, bladder and lung cancer [15,18,19,20]. It is assumed that these animals are detecting odor signatures released from the urine of affected patients [20,21,22,23]. These odor signatures are thought to be volatile organic compounds (VOCs) which can be generated from the human body and released through breath, blood, skin, tissue, urine, and feces [24]. These VOCs could reflect the physiological and metabolic status of the individual [25,26,27]; thus, in individuals with cancer, urinary VOCs could represent the byproducts of the tumor metabolism [28]. Research has been conducted to explore the relationship between the body’s VOC signatures and cancer. Including control groups representing various cancer types would allow for the assessment of biomarker specificity, helping to determine whether they are truly unique to ccRCC or reflect broader cancer-related changes [29]. In general, ccRCCs are epithelial tumors in contact with the urinary space [30,31], making this cancer well suited for a urinary VOC metabolomic approach for biomarker discovery and diagnoses.

Research has shown that VOCs can be detected using several analytical techniques [32,33,34]. Small-molecule biomarkers in urine hold great promise for the early detection and monitoring of renal cancer. These biomarkers, including metabolites, lipids, and volatile organic compounds (VOCs), are products of altered cellular processes in cancerous tissues. Renal cell carcinoma (RCC), the most common form of kidney cancer, often progresses without early symptoms, making the identification of non-invasive biomarkers particularly valuable. Urine, in direct contact with the kidneys, provides a rich source of potential diagnostic information. Researchers are increasingly focusing on profiling these small molecules to distinguish renal cancer patients from healthy individuals and those with other conditions, including gold nanoparticles-assisted laser desorption/ionization mass spectrometry (GALDI-MS) or nuclear magnetic resonance (NMR). Advances in metabolomics and other analytical technologies have enhanced the ability to identify specific biomarker signatures, which could lead to earlier detection, more personalized treatment approaches, and improved patient outcomes [32,35,36,37,38].

Using a gas chromatography/mass spectrometry (GC-MS) approach, our previous study identified a prostate cancer-specific urinary VOC profile in men diagnosed by transrectal ultrasound-guided prostate biopsy [39]. In addition, our studies were able to distinguish men presenting with low- versus high-risk diseases. One study by Noriega Landa et al. explored fatty acids (FAs) as potential biomarkers for prostate cancer (PCa) detection, proposing a urinary FA-based model. Through analysis of urine samples from 334 biopsy-designated PCa positive and 232 biopsy-designated PCa negative subjects, a final FA model was developed, showing higher accuracy (AUC = 0.71) compared to the PSA model (AUC = 0.51), suggesting urinary FAs as a promising non-invasive alternative for PCa diagnosis [40]. Badmos et al. used metabolomics to investigate urine samples from 386 male adults, resulting in the development of a model with a 0.88 AUC for PCa diagnosis and an average 0.78 AUC for distinguishing between low-grade and intermediate/high-grade PCa, offering promising advancements in PCa screening and assessment to address challenges related to over-diagnosis and over-treatment [41].

The knowledge gap regarding the use of VOCs for renal cancer diagnosis stems from insufficient research on the distinct VOC signatures associated with renal cancer compared to other diseases. Additionally, there is a lack of large-scale validation studies to confirm the reliability and accuracy of VOC-based diagnostic models in detecting renal cancer. Urine is an ideal biospecimen for ccRCC detection due to its direct contact with the renal system, potentially containing specific biomarkers indicative of renal pathologies. Its non-invasive collection method makes urine particularly advantageous for routine screening and monitoring of ccRCC progression. As ccRCC is the most common subtype of RCC, this study initiated an exploratory experiment where we focused on detecting urinary VOCs for ccRCC diagnosis. Using the well-established methodology developed in our laboratory, this study was to develop a urinary VOC-based diagnostic model that could discriminate ccRCC patients from healthy controls.

## 2. Materials and Methods

### 2.1. Study Design

Approval was obtained from the Institutional Review Board (UTEP and Geisinger) and written informed consent was obtained from all patients. For ccRCC diagnostic model development, 276 total urine samples were obtained from (a) preoperatively from 233 pathologically confirmed ccRCC patients on the day of surgery (partial or radical nephrectomy) and (b) 43 self-reported healthy control patients with no evidence of cancer. Control patients consisted of patients with benign urologic complaints without history of malignancy, polypharmacy, comorbidities (≤2), and who also have available renal imaging within one year of the start of the study that was negative for any solid or complex cystic renal lesions. Subjects with complex renal cysts, benign renal tumors, or history of current or prior non-clear-cell renal malignancy were excluded from the study. To simulate a real-world clinical scenario, there were no diet or urine collection time restrictions.

**Figure 1:**
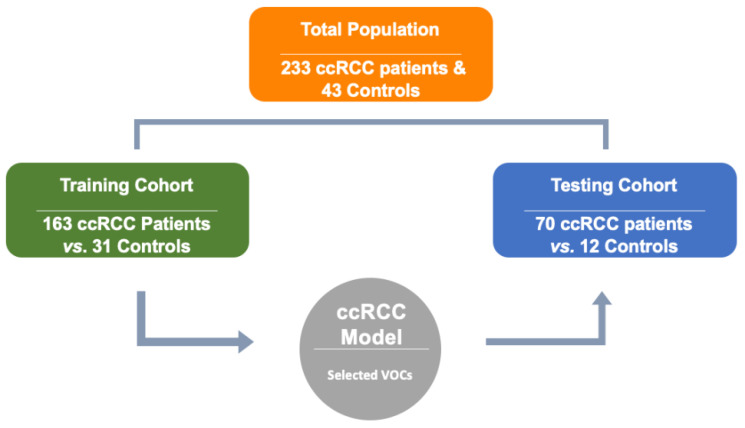
The partitioned total patient population used within training and testing cohorts to generate selected VOCs for diagnostic prediction of ccRCC.

Patients were divided into two groups: a training group (for model development) and a testing group (for model validation). The training set contained urine samples from 163 pathologically confirmed ccRCC patients and 31 healthy controls. For the testing group which was to evaluate the final performance of the ccRCC diagnostic model, 70 ccRCC patients and 12 healthy controls were involved. Figure 1 illustrates the partitioning of the total patient population used to train and test the cohorts. The demographic data of the cohort population is shown in Table 1 (A) and a comparison of the basic characteristics of both the cases (ccRCC patients) and controls is shown in Table 1 (B). While tumor grades were not readily available for all samples in our cohort, our available data encompassed all tumor grades, although most samples did not specify tumor grade data. The aim of this model was to detect ccRCC at all stages. Urine samples of the patients were collected at the medical facilities and laboratory and stored at −80 °C until chemical analyses.

### 2.2. Chemicals and Materials

Mirex (99.0%, Dr. Ehrenstorfer GmbH, Augsburg, Germany), used as the internal standard, was purchased from the Laboratories of Dr. Ehrenstorfer, Germany. Mirex solution of 100 mg·L^−1^ was prepared in methanol (LCMS grade, Burdick & Jackson (Muskegon, MI, USA)). Hydrochloric acid (HCl, 37%) was purchased from Sigma–Aldrich (St. Louis, MO, USA). Ultra-pure deionized water from Milli-Q system (Millipore, Bedford, MA, USA) was used in the preparation of HCl solution and dilution of urine samples.

### 2.3. Extraction and Chemical Analysis of VOCs from Urine Samples

Urine samples were processed through centrifuging and stir-bar sorptive extraction as described previously [39,40]. Briefly, VOCs from urine samples were analyzed in a thermal desorption unit, TDU (Gerstel, Mülheim, Germany), coupled with a GC/MS system (6890/5973-N GC/MS, Agilent Technologies, Wilmington, DE, USA). The National Institute of Standards and Technology (NIST) Library NIST17 was used for the identification of VOC profile in urine samples. All samples were analyzed in a blinded and coded fashion during the instrument analyses [39].

Quality control (QC) samples were prepared using 20 mL of ultra-pure deionized water with the same amount of mirex internal standard (300 uL of 1 ppm) as that added to each urine sample. The QC samples were analyzed after every 20–30 urine sample runs to monitor instrument performance and data consistency.

### 2.4. Data Processing and Statistical Analysis

A detailed account of the performed analyses has been previously published [39]. The urinary VOCs were identified by the library NIST17 according to the matching quality of the MS spectra produced by the instrument. We implemented a filter to a matching quality of 50% or greater to ensure satisfactory VOC identification in further data processing. The relative intensity of each VOC peak was then normalized against that of mirex to enable semi-quantitative analysis of VOCs in the statistical analysis (area ratio). MetaboAnalyst 5.0 was used to generate a partial least squares discriminant analysis (PLS-DA) plot to visually represent the definitive clustering between the ccRCC-positive and healthy cohorts. The dataset was pre-processed by an interquartile range (IQR) of 40% filtered out, then a log transformation (base 10) was applied to normalize the response variable (area ratio). The pre-processed data developed a model to identify a linear combination of the target VOCs which show the greatest discrimination between cohorts. The PLS-DA was then used to generate a variable importance in projection (VIP) plot to identify VOCs which showed a greater discriminatory power.

The statistical significance of each VOC was assessed using the Wilcoxon rank-sum test. Heat maps were generated to visualize significant VOCs (*p* < 0.05) among the ccRCC-positive and control groups; a comprehensive list of the significant VOCs are shown in the Supplementary Table S1. Using a liberal cutoff (*p* < 0.2), a larger set of VOCs was selected to develop a logistic regression model for further identification of noteworthy VOCs. The VOC-based diagnostic tool was developed using logistic regression [42]. The final logistic model was evaluated using the Receiver Operating Characteristic (ROC) curve, and its performance was measured based on jackknife prediction [42,43,44]. Jackknife cross-validation was to strengthen the robustness of our findings and to estimate variability and prevent overfitting, which further validates the performance of the model despite the limited control sample size. All the analyses were performed using the open-source statistical computing package R (version 4.2.2) and MetaboAnalyst 5.0 platform [45,46].

## 3. Results

**Figure 2:**
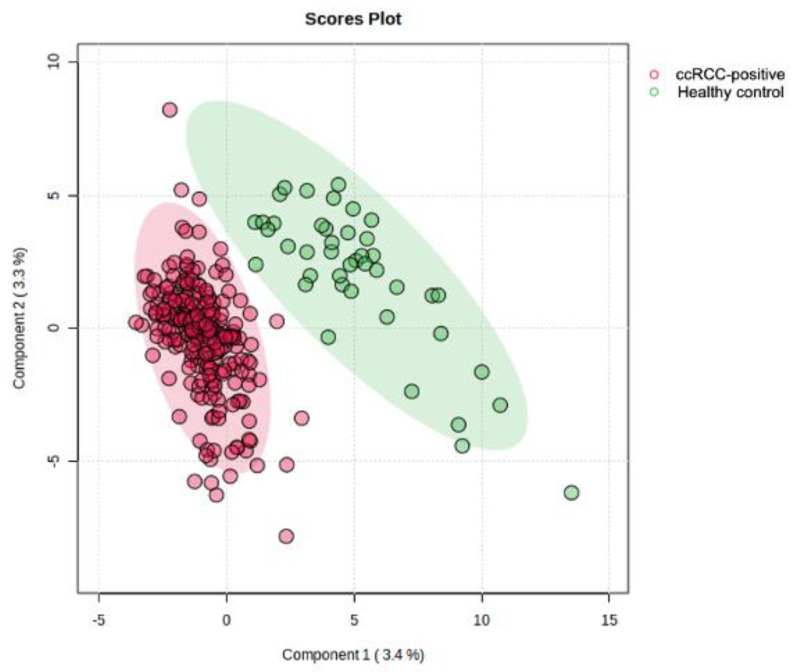
Partial least squares discriminant analysis plot (PLS-DA) comparing the urinary VOCs detected in ccRCC and healthy control cohorts.

All VOCs were identified based on their occurrence and relative quantity in the urine samples. The relative quantity of each VOC was determined after normalization to mirex. Mirex was selected as the internal standard (IS) because it is absent in human urine, eliminating any interference with the urinary VOC profile. A total of 6218 potential VOCs were detected in urine collected from the training cohort. Figure 2 is the partial least-squares discriminant analysis plot (PLS-DA) used as a supervised classification method to describe, predict, and discriminate variable selection. As shown, both ccRCC and healthy control cohorts showed distinction between one another indicating the potential for cohort-specific VOCs to be identified via modeling.

**Figure 3:**
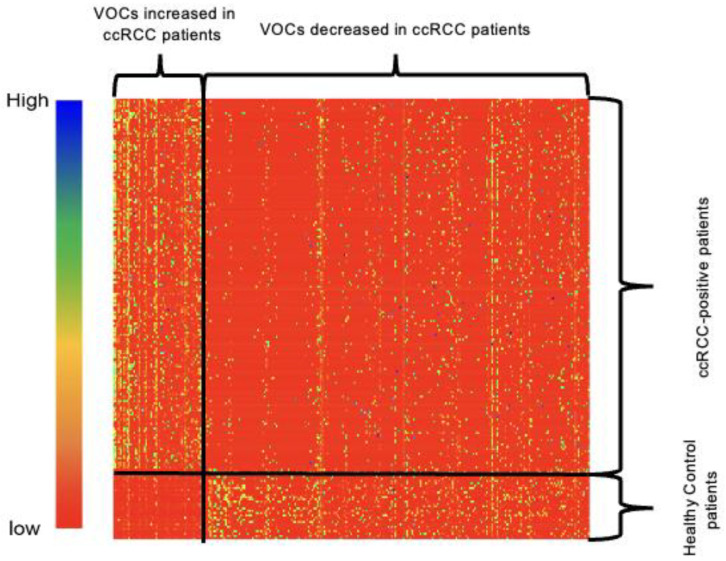
Heat map of significant VOCs in clear cell renal cell carcinoma (ccRCC) vs. controls samples by Wilcoxon test (*p* < 0.05). 56 VOCs were predominant in the cancer group urine samples and 227 VOCs were elevated in the controls. The correlation between VOCs and patients ranges from low (red) to high (blue).

Among the 6218 potential VOCs detected in urine collected from the training cohort, we further used Wilcoxon rank sum test at statistical significance *p* < 0.05 and identified 56 VOCs were predominant in the cancer group urine samples and 227 VOCs for the controls. A heat map was generated to visualize the distribution of those significant VOCs in patients is shown in Figure 3. The comprehensive list of the 283 VOCs is included in Supplementary Materials Table S1.

**Figure 4:**
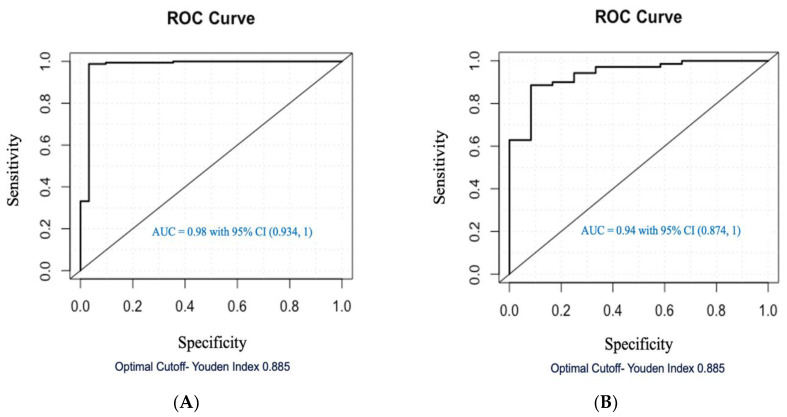
(**A**) The ROC curve for VOC ccRCC diagnosis logistic model verified in the training group with 194 patients (163 ccRCC vs. 31 healthy control). (**B**) The ROC curve for VOC ccRCC diagnosis logistic model validated in the testing group with 82 patients (70 ccRCC vs. 12 healthy control).

To develop the regression diagnostic model, a boarder range of VOCs were selected using *p* ≤ 0.20. After further selection with *l*_1_ regularization, the final logistic model selected 24 VOCs (Table 2). The performance of the ccRCC diagnostic model was assessed using the training set of 163 ccRCC patients and 31 healthy controls. Based on predicted probabilities from the final model via jackknife cross-validation, the area under the receiver operating characteristic curve (AUC-ROC) was 0.98 with a confidence interval 0.934 to 1 with a 99% sensitivity and 97% specificity (with cutoff point of 0.885 obtained by Youden Index) (Figure 4A). The use of jackknife cross-validation helps to reduce bias, estimate variability, and prevent overfitting, thereby providing a more reliable assessment of the model’s discrimination power between VOCs in the urine of ccRCC patients and healthy controls. To validate the performance of the developed ccRCC diagnostic model, a separate cohort of patients was used as the testing group. Via Jackknife prediction, the AUC was 0.94 with a confidence interval of 0.874 to 1 (Figure 4B). Compared to the training cohort, the testing cohort had 86% sensitivity and 92% specificity (with an optimal cutoff point of 0.885). These results support the discrimination power of urinary VOCs in ccRCC diagnosis. In the final logistic model, 24 VOCs were selected (Table 2). Those 24 VOCs selected for the RCC screening model include alkanes, ketones, esters, and alcohols. Among the 24 VOCs, 14 dominated the healthy control cohort and 10 dominated the ccRCC-positive cohort (shown in asterisk/bolded).

**Figure 5:**
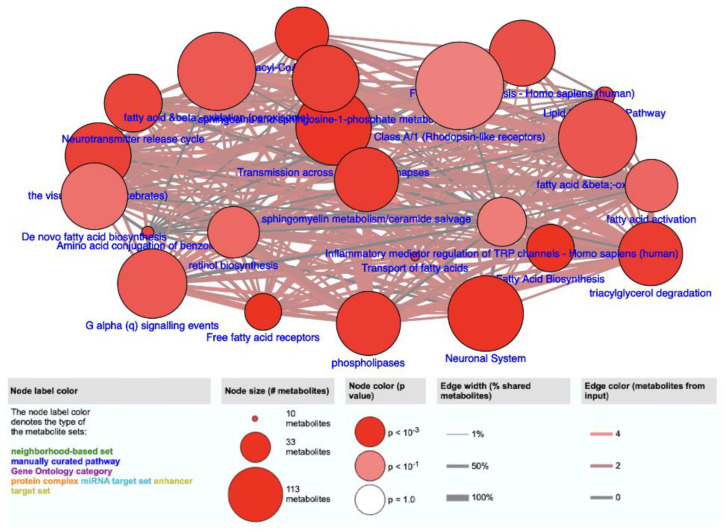
Visual representation of 23 biological pathways generated from the 283 significant VOCs found in the training cohort by Wilcoxon rank-sum test with *p* < 0.05.

In order to better understand the role of the selected VOCs in biological pathways, we used the metabolite analysis in ConsensusPathDB (http://cpdb.molgen.mpg.de/ (accessed on 9 August 2023)) to study the 283 urinary VOCs that were selected by their significance (*p* < 0.05) in distinguishing ccRCC patients from healthy controls. These 283 significant urinary VOCs were found to be linked to several pathways related to amino acids, lipids, fatty acids (Figure 5, Table 3). We do not know the definitive trigger for metabolic dysregulation, but we hypothesized that these compounds may be the result of peroxidation hydrolysis of lipids and fatty acids.

## 4. Discussion

This study was designed to evaluate the clinical utility of the urinary VOCs metabolomic approach to diagnose ccRCC. With a total of 276 ccRCC and healthy control urine samples and using logistic regression, a ccRCC-diagnostic model including 24 VOCs was developed and evaluated with an AUC of 0.98 in the training cohort (sensitivity: 99% and specificity 86%) and an AUC of 0.94 in the testing cohort (sensitivity: 86% and specificity 92%). Table 4 shows a brief comparison of the prediction performance of other studies in the literature, including both metabolomic and proteomic approaches, and this study.

In a previous study, Monteiro et al. developed and optimized a headspace-solid phase microextraction sampling coupled with a gas chromatography/ion trap/mass spectrometry method to study the volatile human urinary metabolome in RCC patients [27,50]. Using an unsupervised principal component analysis (PCA), the research demonstrated the ability of urinary VOCs to discriminate between RCC patients and healthy controls. In the study of 30 RCC patients and 37 controls, the researchers identified 21 discriminatory VOCs using the PCA model [27]. However, after two internal independent studies, only 2 VOCs (2-oxopropanal and 2,5,8-trimethyl-1,2,3,4-tetrahydronaphthalene-1-ol) were validated. In our ccRCC-specific study, we found 10 VOCs which dominated the ccRCC cancer cohort, while 14 VOCs were detected higher in the healthy control cohort. However, the two VOCs reported in Monteiro’s study were not detected in our list (Table 2 and Table 4).

In another study, Wang et al. tested the urine specimens of 22 RCC patients pre- and post-operatively and compared them to those of 25 healthy controls [47]. Using PCA, partial least-squares discriminant analysis (PLS-DA) and two-sided *t*-tests, distinct VOCs were selected based on a variable importance in the projection (VIP) value > 1.2 and a similarity threshold of 75% using the NIST 11 database for VOC screening quality. RCC patients were found to have increased levels of phenol; decanal;1,6-dioxacyclododecane-7,12-dione; 1-bromo-1-(3-methyl-1-pentenylidene)-2,2,3,3 -tetramethyl-cyclopropane; nonanal; 3-ethyl-3-methylheptane; isolongifolene-5-ol; 2,5- cyclohexadiene-1,4-dione, 2,6-bis(1,1-dimethylethyl); tetradecane; aniline; and 2,6,10,14- tetramethyl-pentadecane and decreased levels of styrene; 4-heptanone; and dimethyl silanediol. These metabolites were linked to lipid oxidation and oxidative stress [47]. Furthermore, preoperative patients were compared to postoperative patients, and a unique set of three VOCs were identified. These include elevated levels of 2-ethyl-1-hexanol and cyclohexanone and decreased levels of 6-t-butyl-2,2,9,9-tetramethyl-3,5-decadien-7-yne in the preoperative patients. The discrepancy between the VOCs identified in the RCC versus control cohort and the pre- and post-operative RCC cohort are unclear, particularly because no independent validation in a new cohort of patients was conducted. That said, it raises the question about what constitutes a more reliable comparative group for identifying RCC-specific urinary VOCs, RCC patients versus healthy controls or the same patient pre- and post-treatment. In our study, 2-ethyl-1-hexanol was also found to be significantly more dominant in ccRCC patients’ urine sample. We also found three cyclohexanol-related compounds which were dominant in healthy controls.

Amaro et al. set out to explore the clinical need for RCC early detection [16]. The premise of the experiment focused on the altered metabolic pathways that have the potential to discriminate RCC subtypes of clear cell, papillary, and metastatic in six different cell lines. Using GC-MS coupled with multivariate and univariate analyses, the results suggested that ketones, alcohols, alkanes, and aldehyde groups played an important role in the discrimination. The metabolite panel consisted of significant alterations in cyclohexanone, acetaldehyde, cyclohexanol, decanal, decane, dodecane, and 4-methylbenzaldehyde within the metastatic RCC cell lines. Interestingly, the group also emphasized that there was a significant increase in 2-ethylhexanol in RCC cell lines compared to the normal cell lines; this phenomenon was attributed to the hydroxylation of various reactive oxygen species (ROS) during mediated lipid peroxidation [16]. This finding supports our study where 2-ethylhexanol showed the greatest significance between control and RCC patients. Even though the role of 2-ethylhexanol in RCC is beyond the scope of this study and more validation is necessary, 2-ethylhexanol could be used as a potential biomarker for noninvasive urine-based detection. In addition, many alcohols, ketones, and alkanes were also elected in our RCC diagnostic model (Table 2), supporting the reported findings.

In addition to organic metabolites as RCC biomarkers, researchers have explored using tumor specific protein for RCC diagnostic and screening biomarkers. Morrissey et al. targeted the screening biomarkers aquaporin-1 (AQP-1) and perilipin-2 (PLIN2) to detect and diagnose ccRCC and papillary renal cell carcinoma (pRCC) [48]. Urine samples were obtained from 720 patients in for routine abdominal CT screening, 80 healthy volunteers, and 19 patholically confirmed RCC and the biomarkers concentrations were determined by ELISA and Western blot. Concentrations of AQP-1 and PLIN-2 were found to be 12 times higher in the RCC cohort compared to the healthy and screening cohorts. The combined AUC-ROC for the urinary AQP-1 and PLIN-2 detection was greater than 0.99 with a sensitivity greater than 95% and specificity greater than 91% indicating the feasibility of AQP-1 and PLIN-2 as potential RCC screening biomarkers. This study highlighted the potential of using tumor-specific proteins as diagnostic and screening biomarkers for RCC. Mijuskovic et al. addressed the infamous kidney injury molecule-1 (KIM-1) and AQP-1 as potential urinary biomarkers for ccRCC early detection [49]. Urine samples were collected from 40 healthy and 40 renal tumor-positive patients and analyzed using commercially available ELISA kits. The group conducted a comparative study between clinical and pathological characteristics in healthy volunteers, pre-operative, and post-operative patients. Results demostrated that patients with higher grade tumors had an elevated level of urinary kidney injury molecule-1 (uKIM-1) compared to low-grade lesions. A comparison of urinary aquaporin-1 (uAQP-1) indicated there were no significant correlations between pre-operative concentrations, grade and stage, and tumor size. In summary, uKIM-1, but not uAQP-1, was significantly elevated in patients with cRCC compared to healthy subjects. The overall results did not support the aforementioned Morrissey et al. study [48] but suggested that uKIM-1, due to its noninvasive sampling, simplicity, and support by the literature and commercial ELISA kits, can serve as a valuable and reliable biomarker for cRCC diagnosis and postoperative monitoring in routine clinical practices [48]. Although the performance of urinary protein markers seems impressive, the process of protein extraction is time consuming making their application in cancer screening less attractive.

In this study, our ccRCC-specific urinary VOC model was developed based on the 24 VOCs distributed in the urine of cancer patients as compared to the controls. Several VOCs display biological significance. For instance, among the 24 VOCs identified in this study (Table 2), the long carbon chain and/or carbonyl group of Heptadecanolide; 2-Ethylhexyl methyl isophthalate; Cyclohexanol, 5-methyl-2-(1-methylethyl)-; 1-Propene-1,2,3-tricarboxylic acid, tributyl ester; Cyclohexane, 1,3,5-triphenyl-; Benzene, 1-methyl-4-(1,2,2-trimethylcyclopentyl)-, (R)-; cis-Vaccenic acid; Cadala-1(10),3,8-triene; 1,4-Bis(trimethylsilyl)benzene; l-(+)-Ascorbic acid 2,6-dihexadecanoate; Cyclohexene, 6-ethenyl-6-methyl-1-(1-methylethyl)-3-(1-methylethylidene)-, (S)-; and 2-Methyl-6-(p-tolyl)hept-2-en-4-ol support their role in amino acid conjugation, hydrolysis, and peroxidation of fatty acids [40,51,52,53]. Additionally, 2-ethyl-1-Hexanol is a peroxidation product of oleic acid (C_18_H_34_O_2_), a (poly) unsaturated fatty acid with potent anti-inflammatory properties [16,54,55]. The literature has warranted 2-ethyl-1-Hexanol as a potential VOC that has been detected in urine for prostate and renal cancer. Interestingly, our research also detected 2-ethyl-1-Hexanol dominating in ccRCC patients, thus validating the previous literature. It should be noted that this metabolite could be derived from diethyhexyl phthalate (DEHP), which has the potential to induce apoptosis, overall leading to renal cancer progression [56].

Three benzene derivative VOCs, Benzene, 1-methyl-4-(1,2,2-trimethylcyclopentyl)-, (R)-; Benzene, 1,2-dichloro-4-methyl-; and Benzeneethanamine, N-methyl-, were selected in discriminating ccRCC from healthy controls. Benzene and its derivatives have been classified as carcinogens based on their metabolic breakdown leading to reactive intermediates; these metabolized compounds pose the greatest toxicity to the liver [57,58,59]. Exposure is commonly encountered in industrial and occupational settings and has been linked to an increased risk of cancer, particularly bladder cancer [60]. Benzene and some benzene derivatives have been associated with an increased risk of cancer, especially leukemia [61]. This connection is primarily due to its potential to undergo metabolic activation, leading to the formation of reactive intermediates that can bind to DNA and cause genetic mutations [62]. However, for reasons unknown, the benzene-related compounds identified in this study were dominant in the control group.

As the significant VOCs identified in this study (Table S1) were found to be involved in metabolic pathways, we further discussed some of the metabolic pathways to bring insight into how these pathways could be linked to cancer. The preliminary pathway study shown in Figure 5 and Table 3 indicated the involvement of VOCs in fatty acid biosynthesis, hydrolysis, and degradation pathways. Ultimately, the involvement of VOCs in these biological processes likely stems from their role as intermediates or byproducts in metabolic pathways. Fatty acid metabolism and lipid metabolism are broad categories of metabolic pathways that encompass the breakdown, synthesis, and modification of compounds with long hydrocarbon chains. In these pathways, alcohols and ketones can undergo oxidation, reduction, and other reactions to participate in the production of energy or the synthesis of cellular components [63]. Fatty acid activation is a crucial step in the utilization of fatty acids for energy, and it ensures that the fatty acids are in a form that can be further metabolized. Different types of fatty acids can be activated and used in various metabolic processes, depending on the energy needs of the cell or tissue. The Literature has not been directly associated with a specific metabolic pathway as it relates to cancer; however, there are implications that it can potentially be involved in various metabolic processes that are associated with the metabolism of fatty acids, lipids, and alcohols in living organisms [10,64,65,66].

Acyl-CoA hydrolysis is a cellular process that is involved in the breakdown of fatty acids for energy production and lipid metabolism. Acyl-CoA hydrolysis is a metabolic process that involves the enzymatic cleavage of acyl-CoA compounds, which are important intermediates in fatty acid metabolism. Although acyl-CoA hydrolysis itself is not directly linked to cancer, alterations in fatty acid metabolism, which includes processes like acyl-CoA metabolism, have been associated with various types of cancer [67,68,69]. Specifically, cancer cells often exhibit changes in fatty acid metabolism to support their rapid growth and energy needs. Some of the key alterations in fatty acid metabolism associated with cancer include increased fatty acid synthesis, enhanced lipolysis (breakdown of stored fats), and alterations in the composition of cellular membranes. Among the 24 VOCs selected by the ccRCC model, two fatty acid-related compounds were selected (acetic acid, hexyl ester and 2-Heptenoic acid, octadecyl ester). While fatty acid metabolism and associated pathways are crucial for normal cellular functions, their involvement in renal cancer still warrants further investigation.

Triacylglycerols (also known as triglycerides) are a type of lipid molecule that serves as a storage form of energy in the body [10,65]. The degradation of triacylglycerols is an essential metabolic process that releases fatty acids for energy production. The relationship between free fatty acid receptors (FFARs) and renal cancer is an area of interest due to the direct link between FFARs and the development or progression of renal cancer [51,52]. FFARs are a class of G protein-coupled receptors (GPCRs) that are involved in mediating various cellular responses to fatty acids. FFARs, such as FFAR1 (GPR40), FFAR2 (GPR43), and FFAR3 (GPR41), play important roles in regulating metabolic and immune responses to fatty acids, with direct linkage to lipid metabolism and immune responses [52]. These receptors are commonly found in various tissues, including the kidneys, making these receptors significant in fatty acid metabolism and signaling which can influence cancer cell behavior and tumor progression [53,67].

The metabolism of sphingosine and its derivative, sphingosine-1-phosphate (S1P), is an area of ongoing research in the context of cancer, including renal cancer. Sphingolipid metabolism includes the interconversion of sphingosine and S1P and is known to play a role in various cellular processes, including cell growth, apoptosis, and cell migration. Sphingomyelin and ceramide are bioactive lipids that play important roles in various cellular processes, including cell signaling, apoptosis (programmed cell death), and inflammation within the metabolic pathway of sphingomyelin metabolism and ceramide salvage [70]. Ceramide and related lipid pathways are critical for normal cellular function. The ceramide salvage pathway involves the recycling of ceramide, which can be generated as a result of sphingomyelin hydrolysis, into other sphingolipids or used as a signaling molecule. Ceramide itself has been implicated in various cellular processes, including apoptosis, regulation of cell growth, and survival [71,72]. There is growing interest in this field for several reasons: (1) S1P receptors act as a ligand for a family of G protein-coupled receptors known as S1P receptors (S1PRs). Activation of these receptors can influence various cellular responses, including cell proliferation, angiogenesis, and immune cell trafficking. Aberrant activation of S1PRs has been associated with cancer development and progression in several types of cancer. (2) In cancers, including renal cancer, angiogenesis is essential for tumor growth and metastasis. S1P may influence angiogenesis through its receptors and downstream signaling pathways. (3) Cell migration is important for cancer cell invasion and metastasis. The balance between S1P and ceramides (another class of sphingolipids) in the cell can affect cell migration. Lastly, (4) Sphingosine is a precursor of S1P and has been associated with apoptosis. Dysregulation of sphingosine metabolism may affect apoptosis and contribute to tumor development [70,71,72,73].

The amino acid conjugation of the benzoic acid pathway or glycine conjugation is a detoxification process in the liver that helps eliminate xenobiotics (foreign or harmful compounds) from the body. Benzoic acid is one of the compounds that can be conjugated with glycine to form hippuric acid, which is then excreted in the urine [60,74]. This process helps the body rid itself of potentially toxic substances. Benzoic acid has been found in food additives/preservatives and has been regulated by many health agencies due to the potential formation of the known carcinogen benzene [60,61,75]. Although amino acid conjugation is a liver metabolic process, it still plays a role in detoxification. The study of amino acids as it relates cancer has been proven beneficial in the development of targeted cancer therapies and possible biomarkers, indicating cancer progression [57]. However, the complexity of amino acids has the potential to be detrimental in its relationship to cancer, where they are essential nutrients for cancer cells, supporting synthesis and proliferation, and also play a role in the immune response, which can ultimately result in metabolic reprogramming [76,77]. In this study, we used GC-MS as our primary analytical technique. Thus, we were not able to detect amino acids in urine. Nonetheless, the significant VOCs found in urine samples were linked to the amino acid conjugation of the benzoic acid pathway, implying that the urinary metabolites detected in this study presented values to further understand the mechanisms of cancer progression.

G alpha (q) signaling events have often been linked with cancer signaling events. G protein-coupled receptors (GPCRs) are a large family of cell surface receptors and they transmit signals into the cell through the activation of G proteins. Signaling stimulates adenylate cyclase to increase cyclic AMP (cAMP) levels, leading to the activation of protein kinase A (PKA). Dysregulation of the G signaling can be implicated in various cancers, thus causing mutations in the guanine nucleotide binding protein and alpha stimulating (GNAS) gene encoding for the Gs alpha subunit, which is commonly associated with cancers: pituitary adenomas, ovarian, and pancreatic tumors [78,79,80]. The signaling of the Gi protein inhibits adenylate cyclase, leading to decreased cAMP levels, and Gq receptors activate phospholipase C (PLC), then promoting the generation of inositol trisphosphate (IP3) and diacylglycerol (DAG), thus causing mutations. Overall, Gi promotes cell survival and reduces apoptosis [80].

There is a complex inter-relationship between sphingosine and sphingosine-1-phosphate metabolism, G alpha (q) signaling events, and Class A/1 pathways. Class A/1 (“Rhodopsin-like receptors”) are a subgroup of GPCRs and are involved in various signaling events [81,82]. Many GPCRs in this class are associated with cancer, as they regulate key cellular processes, including cell growth, differentiation, and migration. The signaling of GPCRs activates the Rho family of guanosine triphosphate hydroxylase enzymes (GTPases), while dysregulated signaling has been linked to cancer metastasis and invasion in various tumor types. Beta-adrenergic receptors (β-ARs) have been shown to respond to stress at the surface of various cells. Stimulation of β-ARs can activate the cAMP pathway, leading to the activation of PKA and other downstream effectors [83]. Therefore, β-Ars and S1PRs have been associated with cancer proliferation and metastasis as seen in breast, ovarian, and colorectal cancer [79,80,82,83]. The specific effects of Class A/1 GPCRs in cancer can vary depending on the receptor subtype and the tumor type, indicating that the activation or inhibition of these receptors may have different consequences in different cancers. Research into the roles of GPCRs in cancer is ongoing, and these receptors are considered potential therapeutic targets in cancer treatment [84,85].

Retinol, or vitamin A, is essential for various biological processes by maintaining healthy vision, regulating gene expression, and supporting immune function. Its metabolism is strictly controlled and involves several enzymes and transport proteins. While retinol itself is not directly related to cancer, deficiencies or excessive intake of vitamin A can adversely affect its metabolism and can impact cancer risk. They are particularly obtained in dietary forms, where the body converts carotenoids to retinol via biosynthesis in the liver and tissues. While retinol/carotenoids have been implicated in various physiological processes, the direct relationship between retinol synthesis and renal cancer is not well established. However, retinol has been linked to skin and lung cancer and has also been targeted as a therapeutic agent [86,87].

In the context of cancer, the relationship between inflammatory mediator regulation of transient receptor potential (IMRTRP) channels in humans is a complex and evolving area of research. TRP channels have been primarily associated with sensory physiology and various pathological conditions including pain, neuroinflammation, and inflammation-related diseases. It is interesting to note that ccRCC urinary VOCs via pathway analysis selected the IMRTRP pathway; although it is not directly linked to cancer, the inflammation response to cancer proliferation influences the activation of the TRP channel [88]. Therefore, IMRTRP has been researched for cancer by exploring the interactions between TRP channels and inflammatory mediators which can potentially lead to new therapeutic strategies for managing cancer-related symptoms and improving cancer treatment outcomes. However, more research is needed to fully grasp the implications of this relationship [89].

The value of other “omics” approaches versus metabolomics to identify ccRCC-specific biomarkers is unclear. In a recent tissue-based metabolic flux profiling study comparing normal renal tissue and adjacent tumors in 138 ccRCC patients, broad shifts in central carbon metabolism, one-carbon metabolism, anti-oxidant response, and increases in glutathione and cysteine/methionine metabolism pathways were observed in patients with progressive and metastatic disease [90]. Interestingly, there was no correlation between metabolic genes expression and corresponding metabolite levels in the tissue.

The heterogeneity of molecular alterations within and between ccRCC patients suggests that a panel of metabolites will be needed to account for the diversity of this disease. Therefore, it is very likely our list of ccRCC-specific urinary VOCs could be further expanded. Even though this study shows good discriminatory ability of urinary VOCs to distinguish between ccRCC and healthy patients, it will be necessary to refine and validate our panel of cancer-specific VOCs and in a larger patient population.

## 5. Conclusions

This investigation was designed to evaluate the clinical utility of the urinary VOCs with statistical modeling for ccRCC diagnoses. The findings indicated that the VOC-based ccRCC diagnostic model had favorable sensitivity and specificity, with AUCs of 0.98 and 0.94, and the high sensitivity (99% and 86%) and the specificity (97% and 92%) for the training data set and testing data set, respectively. This investigation supports the ability of urinary VOC-based diagnostic models for early and noninvasive screening ccRCC patients.

There are several limitations in this study. First, our hypothesis was based on the premise that the success of sniffer dogs in discriminating between cancer and controls is through their ability to detect VOCs emanating from urine samples. However, it is possible that VOCs may only represent a subset of volatile compounds being detected. Nevertheless, the ability to discern a difference between cancer and control samples even with our small sample size supports using VOCs as potential cancer discovery biomarkers. Secondly, urinary metabolite production can vary based on the time of the day urine was collected, age, gender, race, diet, medications, underlying chronic diseases, level of physical activity, and living environment. Therefore, standardization of urine collection and processing, as well as controlling as many of the aforementioned variables as possible, may be crucial to any urinary biomarker discovery study seeking to identify disease-specific biomarkers [91,92,93,94,95]. To test the flexibility and friendliness of clinical application, we did not control many of these factors in this study except for the use of urine samples. Thirdly, our sample size needs to be increased in order to capture the heterogeneity in ccRCC tumor biology. Data from The Cancer Genome Atlas for RCC identified multiple mutations in patients with ccRCC, including the VHL, PBRM1, SETD2, KDM5C, PTEN, BAP1, MTOR, and TP53 genes, thereby emphasizing the anticipated metabolomic heterogeneity in ccRCC [90]. Fourthly, it is unclear whether healthy controls represent the better comparative group to the ccRCC cohort for urinary ccRCC-specific VOC biomarker discovery. Perhaps, comparing pre- and post-treatment samples would provide more confidence in the ccRCC-specific urinary VOCs discovered as each patient would act as their own control. That said, there may be other factors beyond ccRCC extirpation that could account for differences in the VOC profile of the pre- and post-operative patient. Fifthly, the low prevalence of RCC makes developing a population-based screening program challenging. However, the ability of our model to discriminate between ccRCC and healthy controls despite our small sample size appears encouraging.

Overall, our study supports the clinical utility of a urinary VOC-based diagnostic model as the biomarker for ccRCC and highlights the need to validate this urinary ccRCC-specific signature in a larger cohort.

## Figures and Tables

**Table 1:** (A) Demographic information of clear cell renal cell carcinoma and renal cancer-negative patients in the VOC ccRCC diagnosis model development. Data are presented as median (interquartile range) for continuous variables and n (%) for categorical variables. (B) A comparison of the age and gender of both the cases (ccRCC patients) and controls.

(A)	Training Cohort (Model Development)		Testing Cohort (Model Validation)	
	ccRCC Group	Control Group	ccRCC Group	Control Group
No.	163	31	70	12
Age	63 (26–87)	46 (22–78)	60 (33–87)	57 (26–73)
Gender				
M	103	14	49	5
F	60	17	21	7
Tumor grade ^1^		N/A ^2^		N/A ^2^
1	20 (12%)		10 (14%)	
2	23 (14%)		9 (13%)	
3	19 (12%)		11 (16%)	
4	10 (6%)		6 (8%)	
Unknown	90 (56%)		34 (49%)	
**(B)**	**Characteristic**	**Control *N* = 43 ^3^**	**Positive *N* = 233 ^3^**	***p*-Value ^4^**
	Age	50 (29, 60)	63 (56, 70)	<0.001
	Gender			0.011
	M	19 (44%)	152 (65%)	
	F	24 (56%)	81 (35%)	

^1^ Pathology/Biopsy Confirmed (Tumor grade); ^2^ N/A: Not applicable; ^3^ Median (Q1, Q3); n (%). ^4^ Wilcoxon rank-sum test; Pearson’s Chi-squared test.

**Table 2:** The 24 VOCs selected by logistic regression models for ccRCC diagnosis prediction.

CAS Number ^1^	Chemical Formula	Chemical Name	Dominating Group	*p*-Value ^2^	Occurrence	
					Cancer (+) ^3^	Control (−) ^4^
* 000104-76-7	**C_8_H_18_O**	**1-Hexanol, 2-ethyl-**	**ccRCC**	**3.07 × 10^−12^**	**140**	**9**
* 005637-97-8	**C_17_H_32_O**	**Heptadecanolide**	**ccRCC**	**1.35 × 10^−1^**	**27**	**2**
1000465-65-6	C_17_H_24_O_4_	2-Ethylhexyl methyl isophthalate	Control	2.22 × 10^−19^	8	21
015356-70-4	C_10_H_20_O	Cyclohexanol, 5-methyl-2-(1-methylethyl)-, (1.alpha.,2.beta.,5.alpha.)-(.+/-.)-	Control	5.76 × 10^−16^	14	21
001490-04-6	C_12_H_22_O_2_	Cyclohexanol, 5-methyl-2-(1-methylethyl)-	Control	1.39 × 10^−13^	1	11
007568-58-3	C_18_H_30_O	1-Propene-1,2,3-tricarboxylic acid, tributyl ester	Control	1.42 × 10^−13^	2	12
028336-57-4	C_24_H_24_	Cyclohexane, 1,3,5-triphenyl-	Control	2.47 × 10^−12^	2	11
016982-00-6	C_15_H_22_	Benzene, 1-methyl-4-(1,2,2-trimethylcyclopentyl)-, (R)-	Control	7.43 × 10^−10^	0	7
000491-02-1	C_10_H_20_O	Cyclohexanol, 5-methyl-2-(1-methylethyl)-, (1.alpha.,2.alpha.,5.alpha.)-	Control	1.68 × 10^−8^	1	7
000075-31-0	C_3_H_9_N	2-Propanamine	Control	2.29 × 10^−7^	0	5
* 000506-17-2	**C_18_H_34_O_2_**	**cis-Vaccenic acid**	**ccRCC**	**1.22 × 10^−6^**	**38**	**19**
* 013151-34-3	**C_11_H_24_**	**Decane, 3-methyl-**	**ccRCC**	**1.29 × 10^−6^**	**14**	**12**
002305-05-7	C_10_H_18_O_2_	.gamma.-Dodecalactone	Control	7.63 × 10^−6^	4	7
1000140-05-6	C_15_H_22_	Cadala-1(10),3,8-triene	Control	2.12 × 10^−5^	3	6
* 004630-07-3	**C_15_H_24_**	**Naphthalene, 1,2,3,5,6,7,8,8a-octahydro-1,8a-dimethyl-7-(1-methylethenyl)-, [1R-(1.alpha.,7.beta.,8a.alpha.)]-**	**ccRCC**	**3.98 × 10^−5^**	**13**	**10**
1000427-45-5	C_5_H_6_O_2_	4-Methylamino-2(5H)-furanone	Control	8.11 × 10^−5^	1	4
* 013183-70-5	**C_12_H_22_Si_2_**	**1,4-Bis(trimethylsilyl)benzene**	**ccRCC**	**1.13 × 10^−3^**	**65**	**18**
* 1000383-15-8	**C_20_H_40_O_3_**	**Carbonic acid, decyl nonyl ester**	**ccRCC**	**6.09 × 10^−3^**	**6**	**5**
000095-75-0	C_7_H_6_Cl	Benzene, 1,2-dichloro-4-methyl-	Control	6.27 × 10^−3^	2	3
000589-08-2	C_9_H_13_N	Benzeneethanamine, N-methyl-	Control	6.77 × 10^−3^	2	3
1000130-20-8	C_5_H_7_N_3_O_2_	l-Guanidinosuccinimide	Control	7.03 × 10^−3^	2	3
* 028474-90-0	**C_38_H_68_O_8_**	**l-(+)-Ascorbic acid 2,6-dihexadecanoate**	**ccRCC**	**1.13 × 10^−2^**	**7**	**5**
* 005951-67-7	**C_15_H_24_**	**Cyclohexene, 6-ethenyl-6-methyl-1-(1-methylethyl)-3-(1-methylethylidene)-, (S)-**	**ccRCC**	**2.66 × 10^−2^**	**9**	**5**
* 038142-57-3	**C_15_H_22_O**	**2-Methyl-6-(p-tolyl)hept-2-en-4-ol**	**ccRCC**	**4.57 × 10^−2^**	**4**	**3**

^1^ Chemical Abstracts Service number (CAS). ^2^ *p*-value: the *p*-value of selected compounds from the Wilcoxon rank-sum test. ^3^ Cancer (+): ccRCC-positive patients. ^4^ Control (−): healthy control patients. * **Bold:** VOC levels dominant in ccRCC-positive patients’ urine.

**Table 3:** The 23 CPDB metabolic interaction pathways generated from the 283 significant VOCs (Wilcoxon rank sum, *p* < 0.05).

Pathway Name	Pathway Source *	*p*-Value
Free fatty acid receptors	Reactome	1.16 × 10^−4^
Fatty acid biosynthesis	SMPDB	1.16 × 10^−4^
Transmission across chemical synapses	Reactome	1.63 × 10^−4^
Neuronal system	Reactome	1.63 × 10^−4^
Acyl-CoA hydrolysis	HumanCyc	2.29 × 10^−4^
Phospholipases	HumanCyc	3.34 × 10^−4^
Triacylgycerol degradation	HumanCyc	3.34 × 10^−4^
Sphingomyelin metabolism/ceramide salvage	HumanCyc	3.34 × 10^−4^
The visual cycle I (vertebrates)	HumanCyc	4.66 × 10^−4^
Sphingosine and sphingosine-1-phosphate metabolism	HumanCyc	4.66 × 10^−4^
Lipid metabolism pathway	Wikipathways	5.92 × 10^−4^
Transport of fatty acids	Reactome	5.92 × 10^−4^
Neurotransmitter release cycle	Reactome	6.28 × 10^−4^
Amino acid conjugation of benzoic acid	Wikipathways	7.38 × 10^−4^
Fatty acid biosynthesis—Homo sapiens (human)	KEGG	1.08 × 10^−3^
Fatty acid β-oxidation	HumanCyc	1.54 × 10^−3^
G alpha (q) signaling events	Reactome	1.62 × 10^−3^
Fatty acid β-oxidation (peroxisome)	HumanCyc	1.62 × 10^−3^
Fatty acid activation	HumanCyc	3.04 × 10^−3^
Retinol biosynthesis	HumanCyc	3.35 × 10^−3^
De novo fatty acid biosynthesis	EHMN	4.74 × 10^−3^
Inflammatory mediator regulation of TRP-channels- Homo sapiens (human)	KEGG	7.25 × 10^−3^
Class A/1 (Rhodopsin-like receptors)	Reactome	8.77 × 10^−3^

* SMPDB—Small Molecule Pathway Database; HumanCyC—Encyclopedia of Human Genes and Metabolism; KEGG—Kyoto Encyclopedia of Genes and Genomes; EHMN—Edinburgh Human Metabolic Network.

**Table 4:** A comparison of VOCs biomarkers detected in RCC urine and cell line.

Reference	Cohort Size	Analytical Methods	Statistical Methods	AUC-ROC (Sensitivity/Specificity)	Selected VOCs or Biomarkers
Monteiro et al. [27]	30 RCC; 37 healthy (RCC urine)	HS-SPME-GC-IT/MS	PCA	ND *	2-oxopropanal and 2,5,8-trimethyl-1,2,3,4-tetrahydronaphthalene-1-ol
Wang et al. [47]	22 RCC; 25 healthy (RCC urine)	UPLC-MS	Welch Two Sample T-Test, Variable Importance in the Projection (VIP Values), PLS-DA	H vs. RCC: 0.702 (76% and 79%); Pre vs. Post: 0.833 (61% and 88%)	phenol, decanal,1,6-dioxacyclododecane-7,12-dione; 1-brom o-1-(3-methyl-1-pentenylidene)-2,2,3,3-tetramethyl-cyclopropane; nonanal; 3-ethyl-3-methylheptane; isolongifolene-5-ol; 2,5-cyclohexadiene-1,4-dione, 2,6-bis(1,1-dimethylethyl); tetradecane; aniline; 2,6,10,14-tetramethyl-pentadecane; styrene, 4-heptanone; dimethylsilanediol; 2-ethyl-1-hexanol; cyclohexanone; 6-t-butyl-2,2,9,9-tetramethyl-3,5-decadien-7-yne
Amaro et al. [16]	RCC cell lines	HS-SPME-GC-MS	PCA and PLS-DA	ND * for entire VOC panel	cyclohexanone; acetaldehyde; cyclohexanol; decanal; decane; dodecane; and 4-methylbenzaldehyde
Morrissey et al. [48]	19 RCC; 80 healthy (RCC urine)	ELISA and Western Blot	One-way ANOVA and Pearson Chi-square Test	1.0 (100% and 100%); 0.99 (100% and 98%)	AQP-1 and PLIN
Mijuskovic et al. [49]	40 RCC; 40 healthy (RCC urine)	ELISA	Smirnov Test and Mann–Whitney Test	ND *	KIM-1 and AQP-1
**Holbrook et al. (this study)**	**233 ccRCC; 43 healthy** **(RCC urine)**	**SBSE-GC-MS**	**Linear Regression**	**0.94 (86% and 92%)**	**24 (Table 2)**

* ND = Not determined.

## Data Availability

The data presented in this study are available upon request from the corresponding/first authors. The data are not publicly available due to institutional policy regarding data protection.

## Supplementary Materials

The following supporting information can be downloaded at: https://www.mdpi.com/article/10.3390/metabo14100546/s1, Table S1. Significant VOCs (*p* < 0.05) selected and used in the pathway analysis. The Chemical Abstracts Service (CAS) numbers were used to indicate each metabolite.
