# Supplementary material for: Detection and Validation of Organic Metabolites in Urine for Clear Cell Renal Cell Carcinoma Diagnosis
Source: Metabolites. 2024 Oct 13;14(10):546. doi: 10.3390/metabo14100546 (PMC11509871; doi:10.3390/metabo14100546)
Supplement: Supplementary file 1 [file metabolites-14-00546-s001.zip › metabolites-3233660-Supplementary.pdf]

Supplementary Material

Table S1. Significant VOCs ($p < 0.05$) selected and used in the pathway analysis. The Chemical Abstracts Service (CAS) numbers were used to indicate each metabolite.

VOC (CAS Number)	Wilcoxon p-value	VOC (CAS Number)	Wilcoxon p-value	VOC (CAS Number)	Wilcoxon p-value
1000465-66-9	2.22E-19	002955-56-8	0.000750118	007694-45-3	0.01215964
015423-57-1	5.76E-16	014167-67-0	0.000803845	1000454-26-6	0.012325187
000100-45-8	1.15E-13	000120-80-9	0.000859449	1000155-85-6	0.012430853
001497-19-4	1.39E-13	023747-45-7	0.000897575	000119-64-2	0.012712997
007604-99-1	1.42E-13	022934-59-4	0.000919553	019250-17-0	0.012746992
028393-07-9	2.47E-12	006976-28-9	0.000937669	000108-94-1	0.012902626
000104-83-6	3.07E-12	003558-24-5	0.000954591	066225-17-0	0.012986204
000629-23-2	2.35E-11	1000245-47-5	0.00096535	000871-71-6	0.01473037
001724-02-3	4.12E-11	000563-16-6	0.000979559	104926-37-6	0.014943781
1000314-99-7	4.12E-11	055520-96-2	0.001074332	003658-80-8	0.015006787
000119-45-9	4.91E-11	013187-99-0	0.001131554	1000222-21-2	0.015355299
000108-95-2	2.06E-10	000590-19-2	0.001167697	053800-02-5	0.015819369
000314-40-9	2.08E-10	1000400-61-6	0.001167697	000629-99-2	0.017037113
016996-12-6	7.43E-10	000059-48-3	0.001167788	055682-89-8	0.01706749
002216-52-6	5.98E-09	017312-77-5	0.001167788	007098-21-7	0.017110613
000483-77-2	8.90E-09	001931-63-1	0.001206238	000541-46-8	0.017641695
001622-58-8	1.24E-08	015097-49-1	0.001227311	1000131-36-2	0.017909172
002719-64-4	1.31E-08	007087-68-5	0.001289528	074663-87-9	0.018060563
002721-22-4	1.31E-08	016493-20-2	0.001289528	000140-66-9	0.018747363
029899-13-6	1.31E-08	000928-49-4	0.001289626	039095-16-4	0.018747904
000491-07-6	1.68E-08	001007-48-3	0.001289626	1000121-76-3	0.018747904
000629-58-3	1.78E-08	000544-76-3	0.001355738	060714-16-1	0.018974727
000142-83-6	2.12E-08	002245-38-7	0.001707809	000131-70-4	0.019342169

007764-50-3	2.12E-08	066660-38-6	0.001737667	000142-62-1	0.020381667
000334-48-5	2.29E-07	000099-02-5	0.001786941	016982-00-6	0.021224813
000120-61-6	2.29E-07	000586-63-0	0.00179225	000111-03-5	0.02129375
000497-25-6	2.29E-07	000065-85-0	0.001924304	000706-14-9	0.021751262
023445-02-5	2.29E-07	137695-20-6	0.002081651	014384-36-2	0.021886885
000075-83-2	2.29E-07	000111-13-7	0.002175956	006502-22-3	0.022033026
000084-75-3	2.32E-07	000104-51-8	0.002319655	001485-75-2	0.022566855
002400-66-0	2.73E-07	1000421-20-6	0.002402034	103495-51-8	0.023265112
1000293-20-9	3.06E-07	000104-61-0	0.002404462	000931-17-9	0.023405249
328019-24-5	3.16E-07	073105-67-6	0.002459259	003750-07-0	0.023405249
005989-27-5	3.84E-07	028749-81-7	0.002466847	1000443-41-7	0.024717412
002136-71-2	7.76E-07	128231-64-1	0.002491903	001193-24-4	0.02471807
051578-80-4	9.45E-07	000141-62-8	0.002492001	1000364-64-2	0.025512788
000506-21-8	1.22E-06	005989-33-3	0.002492001	004536-86-1	0.026118233
013151-43-4	1.29E-06	1000193-22-2	0.002492001	068595-80-2	0.02624793
004542-57-8	1.59E-06	036452-83-2	0.002671357	005953-76-4	0.026619926
004754-26-1	2.23E-06	001120-72-5	0.002694421	101628-22-2	0.028288727
002091-29-4	2.93E-06	1000129-26-2	0.002839227	053084-33-6	0.028299702
000131-18-0	3.48E-06	000626-22-2	0.003049652	1000298-84-7	0.029443748
055162-49-7	3.93E-06	000057-11-4	0.003118059	197523-62-9	0.030107635
1000302-13-3	3.93E-06	000100-42-5	0.003146877	006222-06-6	0.030254312
000104-67-6	3.93E-06	075163-98-3	0.003155407	016096-32-5	0.03089736
000248-96-4	3.93E-06	1000306-07-2	0.003160641	1000406-10-3	0.031264007
000286-08-8	3.93E-06	004937-62-6	0.003167453	078323-99-6	0.031492341
001195-09-1	3.93E-06	001120-21-4	0.003459229	040711-15-7	0.031654665
1000293-49-7	3.93E-06	000629-93-6	0.00370435	002425-54-9	0.03178985
1000348-10-4	3.93E-06	001599-68-4	0.00397233	002922-51-2	0.031898334
1000434-34-9	3.94E-06	053939-27-8	0.003977909	005129-58-8	0.031985256
055429-84-0	4.55E-06	067902-78-7	0.004114982	000141-78-6	0.034320154

086812-61-5	4.79E-06	020129-39-9	0.004126166	007094-26-0	0.034621801
000112-15-2	5.00E-06	028790-86-5	0.00432447	055429-83-9	0.035162683
007786-67-6	5.37E-06	000099-86-5	0.004620894	072439-84-0	0.035466396
000118-91-2	5.68E-06	000099-87-6	0.004743269	1000336-66-8	0.036383936
014371-10-9	6.00E-06	000694-47-3	0.005240368	019144-09-3	0.036582677
022356-34-9	6.00E-06	006165-40-8	0.005290101	959092-08-1	0.037837415
032772-75-1	6.00E-06	001560-81-2	0.005325389	006785-23-5	0.037887069
000112-31-2	6.74E-06	000112-54-9	0.005378468	000111-84-2	0.03814839
071186-25-9	7.57E-06	057597-14-5	0.005422926	000630-04-6	0.038258285
002305-36-4	7.63E-06	000766-39-2	0.006029618	000066-25-1	0.038760189
017852-28-7	7.94E-06	004187-86-4	0.006029618	000115-07-1	0.039832261
000294-62-2	8.43E-06	000088-04-0	0.006029893	002217-45-0	0.039989177
000124-40-3	1.25E-05	001016-09-7	0.006035372	003179-09-7	0.040688959
000104-76-7	1.87E-05	1000383-15-9	0.006093522	019095-24-0	0.041241214
1000140-07-3	2.12E-05	000095-78-3	0.006267698	054299-96-6	0.041872361
000638-53-9	2.58E-05	000097-95-0	0.006440951	000112-55-0	0.042840696
000112-12-9	3.48E-05	001120-88-3	0.006440951	021426-37-9	0.044724927
1000161-37-3	3.60E-05	000112-53-8	0.006513619	1000430-37-3	0.044797803
082366-24-3	3.66E-05	004292-04-0	0.006513619	041929-05-9	0.045030837
004630-20-0	3.98E-05	1000349-92-1	0.006513619	1000316-28-8	0.045059942
000096-88-8	4.12E-05	013205-57-7	0.006513911	038147-00-1	0.045694382
000124-18-5	6.18E-05	000101-73-5	0.006595926	062199-51-3	0.045900637
071186-27-1	6.93E-05	1000223-05-5	0.006626433	000498-15-7	0.046658476
020235-19-2	7.29E-05	000589-40-2	0.00676879	003221-61-2	0.046658476
1000427-70-6	8.11E-05	016731-94-5	0.00676879	007069-41-2	0.046658476
000095-75-0	8.11E-05	1000130-38-8	0.007032597	015840-60-5	0.046696875
006996-15-2	8.55E-05	042779-08-8	0.007305281	1000309-26-8	0.046876732
000100-84-5	9.01E-05	004110-44-5	0.007305601	003055-94-5	0.047038238
1000360-41-8	9.01E-05	001120-25-8	0.007326314	000088-58-4	0.047363138

034246-54-3	9.01E-05	007391-40-4	0.007336944	054833-55-5	0.0475411
043219-80-3	0.00010058	002243-27-8	0.007342771	000765-87-7	0.049321464
000112-44-7	0.000193111	000111-76-2	0.007588075	1000159-37-6	0.049321464
023726-93-4	0.000252055	001653-32-3	0.007588075	000872-55-9	0.049946118
000112-49-2	0.000260511	001167-87-9	0.007598018		
002621-79-6	0.000265542	072747-25-2	0.007661213		
1000315-53-8	0.000381638	000084-69-5	0.008108448		
010541-56-7	0.000407708	002941-78-8	0.008356296		
000484-52-6	0.000513916	000077-94-1	0.00849458		
000112-92-5	0.000560296	000335-45-5	0.008829518		
067801-07-4	0.000584973	000695-06-7	0.009749836		
002719-62-2	0.000619973	000106-24-1	0.010184039		
017454-48-7	0.000637316	000293-96-9	0.010795099		
025117-31-1	0.000665053	065820-56-6	0.011131034		
1000357-25-8	0.000675474	001067-08-9	0.011152036		
000598-98-1	0.000693988	028580-43-0	0.011323749		
018857-03-9	0.000716144	071579-67-4	0.011534114		
074708-73-9	0.000730672	062016-79-9	0.012043824		
